# Rumination, experiential avoidance, and dysfunctional thinking in eating disorders

**DOI:** 10.1016/j.brat.2010.05.009

**Published:** 2010-09

**Authors:** Adhip Rawal, Rebecca J. Park, J. Mark G. Williams

**Affiliations:** Department of Psychiatry, University of Oxford, OX3 7JX, United Kingdom

**Keywords:** Rumination, Avoidance, Thought processes, Eating disorders, Dysfunctional attitudes, Self-focus

## Abstract

The majority of research in eating disorders (ED) has investigated the content of disorder-specific thoughts, while few studies have addressed underlying cognitive-affective processes. A better understanding of processes underpinning ED may have important implications for treatment development. Two studies were conducted that investigated levels of rumination, beliefs about rumination, experiential avoidance, and aspects of schematic thinking in individuals with eating pathology. The latter was assessed with a newly designed ED-Sentence Completion Task (ED-SCT). Study 1 (*N* = 177) examined relations between ED psychopathology and these variables in a student population. Extending this, Study 2 (*N* = 26) assessed differences between patients with anorexia nervosa and healthy control participants. The results showed that ED psychopathology was related to disorder-specific cognitions, experiential avoidance as well as ruminative brooding but not reflection. A follow-up of anorexia nervosa patients indicated that changes in ED psychopathology were associated with changes in dysfunctional attitudes and maladaptive cognitive-affective processes. These findings highlight cognitive processes that may play an important role in the maintenance of eating pathology.

## Introduction

Psychological disorders are characterised by disorder-specific thoughts. Beck’s (1976) cognitive theory of emotional disorders was the first to specifically suggest that different disorders have unique cognitive profiles that reflect specific disorder-related content. Attention is commonly captured by stimuli related to such material (Mathews & MacLeod, 1986; McCabe & Gotlib, 1995) and likely to become subject to further cognitive processing, for example, by dwelling on or attempting to avoid such content, which often leads to the intensification of concerns (Nolen-Hoeksema, 2000; Watkins & Baracaia, 2001). There is now an extensive amount of literature to suggest that repetitive thought is a feature evident across psychopathological states and characterised by similar processes (see Ehring & Watkins, 2008; Watkins, 2008). Indeed, repetitive, abstract and verbal thinking, with little sustained focus on direct experiential states, defines rumination and worry. Rumination and worry are commonly found in disorders such as depression, anxiety, or post-traumatic stress disorder, and evidence suggests that they are causally related to the development and/or maintenance of cognitive and emotional problems (e.g., Ehring, Frank, & Ehlers, 2008; Segerstrom, Tsao, Alden, & Craske, 2000; Watkins et al., 2007). A better understanding of these transdiagnostic processes underpinning a range of psychopathologies, together with disorder-specific content, is central to the development of novel strategies for intervention (Barnard, 2004).

Much work in the field of eating disorders (ED) has focused on disorder-specific thoughts, suggesting that these individuals are particularly preoccupied with thoughts about the control of their eating, weight and shape (Cooper, Wells, & Todd, 2004; Fairburn, Cooper, & Shafran, 2003; Fairburn, Shafran, & Cooper, 1998). Consistent with this, experimental research in ED has verified the enhanced processing and recall of food-, weight- and shape-related stimuli (see Lee & Shafran, 2004). Most cognitive theories of ED propose the presence of dysfunctional cognitive structures (schemas) to account for this cognitive fixation. The term schema refers to a mental representation of knowledge that serves as a model for understanding the self, one’s environment, and other people by influencing the selection and interpretation of information (Dalgleish, 2004). It is theorised that in ED, issues pertaining to eating, weight and shape are a central component of these cognitive structures, thus increasing their accessibility (Garner & Bemis, 1982; Vitousek & Hollon, 1990; Waller, Kennerley, & Ohanian, 2007). Empirical investigations assessing aspects of schematic thinking are still needed (Woolrich, Cooper, & Turner, 2006). Indeed, the Eating Disorder Beliefs Questionnaire (EDBQ; Cooper, Cohen-Tovée, Todd, Wells, & Tovée, 1997) is one of only few measures designed to assess ED-related dysfunctional attitudes. Findings show the presence of dysfunctional assumptions in ED populations (Cooper & Hunt, 1998; Cooper & Turner, 2000; Cooper, Rose, & Turner, 2005), suggesting that cognitions associating the self with eating, weight, and shape control are common and central to these individuals’ sense of self. Such thoughts are likely to underpin further cognitive processing and likely to contribute to and/or intensify ED-symptomatology, in ways similar to other psychological disorders (see above).

Indeed, some theoretical accounts of ED have suggested that transdiagnostic processes may underlie and contribute to the maintenance of cognitive-affective and somatic manifestations (Park & Barnard, 2006; Park, Dunn, & Barnard, under review; Waller et al., 2007; Wolff & Serpell, 1998). These accounts have highlighted the role of rumination and experiential avoidance as maladaptive strategies for regulating affect. However, there has been little empirical research in ED aimed at assessing such processes.

Rumination is defined as conscious thoughts that revolve around common concerns and that recur in the absence of immediate environmental demands (Martin & Tesser, 1996). Nolen-Hoeksema (1991) proposed a conceptualisation of rumination in the context of depressed mood, defining it as thoughts that repetitively focus the individual’s attention on his or her negative feelings and symptoms, their causes, meanings and consequences. There is considerable evidence showing that rumination is associated with a range of negative outcomes in clinical and non-clinical samples of various psychological disorders (e.g., persistence of negative mood and thinking, impairment of problem-solving and concentration; Ehring et al., 2008; Nolen-Hoeksema, Wisco, & Lyubomirsky, 2008; Rimes & Watkins, 2005; Watkins & Teasdale, 2001, 2004).

While it is well recognised that preoccupation with the control of eating, weight and shape is a feature of core ED psychopathology (Cooper & Fairburn, 1987; Fairburn et al., 2003), there are surprisingly few studies examining the process of rumination in ED. In a community sample of female adolescents, the tendency towards rumination in response to low mood predicted the onset of binge eating and increases in bulimic symptoms (Nolen-Hoeksema, Stice, Wade, & Bohon, 2007). Based on interviews, Troop, Holbrey, and Treasure (1998) found higher rates of cognitive avoidance and rumination in response to life events and difficulties in ED patients compared to non ED-controls.

Experiential avoidance refers to a process involving excessive negative evaluations of unwanted internal events such as thoughts, feelings, and bodily sensations, an unwillingness to experience these private events, and deliberate efforts to control or escape from them (Hayes, Wilson, Gifford, Follette, & Strosahl, 1996). As such, there is some overlap with thought suppression, and both avoidance and suppression of internal events are thought to contribute to the persistence of problematic emotional states (Hayes et al., 1996; Purdon, 1999). Avoidant personality traits are common in ED (Diaz-Marsa, Carrasco, & Saiz, 2000), and it has been suggested that these pre-date the onset as well as contribute to the maintenance of abnormal eating patterns (Lyubomirski, Casper, & Sousa, 2001; Troop & Treasure, 1997; Troop et al., 1998). Findings of deficits in detecting emotional and body-state signal support this position (Kucharska-Pietura, Nikolaou, Masiak, & Treasure, 2004; Wagner, Ruf, Braus, & Schmidt, 2003), and ED may even facilitate the capacity for avoiding emotions and bodily states (Serpell, Treasure, Teasdale, & Sullivan, 1999), making this process particularly pertinent to these disorders.

Finally, research in depression has shown that meta-cognitive beliefs, for example, in the form of beliefs about the benefits of rumination are common and proximal factors in determining the frequency and stability of rumination in samples that have a tendency to ruminate (Barnhofer, Kuehn, de Jong-Meyer, & Williams, 2006; Watkins & Baracaia, 2001; Watkins & Moulds, 2005). It is possible that beliefs about rumination act as an internal control mechanism that strengthens ruminative thinking (Flavell, 1979), which may be one factor accounting for its persistence. Strategies to control cognitions (e.g., ‘If I don’t keep thinking about my eating then I’ll lose control and gain weight’) are often observed in clinical work in ED (Cooper, Grocutt, Deepak, & Bailey, 2007). However, no direct assessments of beliefs about rumination have been carried out.

This article reports two studies where rumination, experiential avoidance, and aspects of schematic thinking were directly assessed. Together these studies were particularly aimed at advancing understanding of the processes by which individuals are likely to *react to* ED-concerns.

## Study 1

The aim of Study 1 was to measure levels of rumination, beliefs about rumination, experiential avoidance, and aspects of schematic thinking in a student population. Schematic thinking was assessed using a newly designed Eating Disorder-Sentence Completion Task (ED-SCT), which was based on the Sentence Completion Task (SCT) devised by Teasdale, Taylor, Cooper, Hayhurst, and Paykel (1995) to assess aspects of schematic thinking in depression. In Teasdale et al.’s version, participants were required to complete self-referring sentence stems involving the anticipated outcome of social approval or personal achievement (e.g., ‘If I could always be right then others would ……. me’) with the first word that came to mind. The task was originally designed to pit predictions from a schematic model perspective (Barnard & Teasdale, 1991; Teasdale & Barnard, 1993) against those from Bower’s (1981) associative network theory. According to Bower, negative mood states increase accessibility of all negative concepts and thus would lead to the completion of sentences, such as the one above, with a negative word. In contrast, the schematic model approach proposes that mood states influence thinking by changing the inter-relationship between constructs (in depression implying a close dependence between self-worth and social approval) and thus would lead to completions with a positive word. Results showed that depressed patients gave more positive completions than non-depressed controls, and that changes in mood were associated with changes in the number of positive completions (Sheppard & Teasdale, 1996; Teasdale, Lloyd, & Hutton, 1998; Teasdale et al., 1995). In this way, the task separated a simple valence effect from an effect linked to higher-level meaning. Results from these studies supported the use of this task as a tool for assessing the *presence* and *changes* of schematic thinking in depression.

For the present study, an ED-SCT was developed by minimally adapting sentence structures created by Teasdale et al. in order to reflect the operation of ED-related schematic thinking. Specifically, individuals with ED commonly associate eating, weight and shape with social-, self-acceptance, and control (Cooper et al., 1997), and sentences were designed to tap into such underlying assumptions. Thus, an individual with significant ED-concerns completing a sentence such as ‘If I could always be my ideal weight and shape then others would …… me’ might be more likely to use a positive completion such as ‘like’, ‘accept’ or ‘respect’ than an individual with mild or no ED-concerns. This is because of the closer dependence of the individual’s self-view on success at meeting eating, weight and shape goals, which as described further above, is thought to be a reflection of the centrality of such issues to mental representations (schemas). Overall, 12 sentences that tapped into ED-related assumptions in this way were developed (see Method).

We carried out an online survey to enable recruitment of a relatively large study sample. We hypothesised that ED-symptomatology would be significantly correlated with and predict levels of rumination, beliefs about rumination, experiential avoidance, and the number of positive completions on the ED-SCT.

## Method

### Participants

177 student volunteers from the University of Oxford completed the survey. Participants reported no neurological or psychiatric disorders. The global score of the Eating Disorder Examination-Questionnaire (EDE-Q; Fairburn & Beglin, 1994) was used to measure ED-concerns. The mean age of the sample was 22.39 years (*SD* = 5.13, range = 18–50). 122 participants were female with a mean age of 22.22 (*SD* = 4.53), and 55 were male with a mean age of 22.76 (*SD* = 6.30). Participants’ BMI fell within the normal range (full sample: *M* = 21.95, *SD* = 2.72; Male: *M* = 22.12, *SD* = 2.17; Female: *M* = 21.87, *SD* = 2.93). Participants received book vouchers for participation.

### Measures

Participants were asked to provide information on their gender, age, height, weight as well as details on any ED and/or other psychiatric disorder suffered from currently or in the past.

#### Self-report

The EDE-Q (Fairburn & Beglin, 1994) is a 28-item self-report measure of ED psychopathology based on the Eating Disorders Examination (EDE; Cooper & Fairburn, 1987). The EDE-Q assesses behavioural and attitudinal features of ED over the preceding 4 weeks and comprises four subscales: restraint, eating concerns, weight concerns and shape concerns. Scores on each subscale can range from 0 to 6 and the overall ‘global’ score is the average of subscale scores. The EDE-Q has shown good reliability and validity. Mond, Hay, Rodgers, Owen, and Beumont (2004) report a Cronbach’s alpha of .93 for the global scale. Test re-rest coefficients have been found to range from .81 to .94 for the four subscales in a sample of female university undergraduates (Luce & Crowther, 1999).

The Patient Health Questionnaire (PHQ-9; Kroenke & Spitzer, 2002) is a 9-item scale designed to measure symptoms of depression over the preceding 2 weeks. Total scores can range from 0 to 27. Scores of 5, 10, 15, and 20 represent cut-points for mild, moderate, moderately severe and severe depression, respectively. Good internal consistency (Cronbach’s alpha of .85), concurrent validity with the Beck Depression Inventory (BDI; Beck, Ward, Mendelson, Mock, & Erbaugh, 1961) and the depression subscale of the Hospital Anxiety and Depression Scale (Zigmond & Snaith, 1983), and test–retest reliability (*r* = .89) have been reported (Adewuya, Ola, & Afolabi, 2006; Cameron, Crawford, Lawton, & Reid, 2008).

The Generalised Anxiety Disorder Assessment (GAD-7; Spitzer, Kroenke, Williams, & Lowe, 2006) is a 7-item measure for assessing symptoms of generalised anxiety disorder over the last 2 weeks. Total scores can range from 0 to 21. Scores of 5, 10, and 15 represent cut-points for mild, moderate, and severe anxiety, respectively. Spitzer et al. report a Cronbach’s alpha of .92, a correlation of .72 with the Beck Anxiety Inventory (BAI; Beck, Epstein, Brown, & Steer, 1988), and test–retest reliability of .83.

The ED-SCT (adapted from Teasdale et al., 1995) used to assess ED-related schematic thinking was developed for this study. The task required participants to ‘fill in the blank with the first word that comes to mind to make a sentence’ to the 12 sentence stems which are shown in Table 1 along with typical dysfunctional/functional completions given.

Each completion was scored positive or not, judging exclusively the completion word in isolation, independently of the context provided by the stem. The ratings were made by two independent raters, consistent with the procedure employed by Teasdale and colleagues. Inter-rater agreement in the current study was high, with an overall agreement of 98% between raters and a kappa value of .97. The ED-SCT total score was the number of sentence stems to which a positive completion was made. Scores could thus range from 0 to 12.

The EDBQ (Cooper et al., 1997) is a 32-item questionnaire that assesses assumptions and beliefs associated with ED. Each item is rated on a scale from 0 to 100. The EDBQ consists of four subscales: (a) negative self-beliefs, (b) weight and shape as a means to acceptance by *others*, (c) weight and shape as a means to *self*-acceptance, and (d) eating behaviour as a means to control. Studies showed good reliability (Cronbach’s alphas for each factor ranged from .86 to .94) and construct validity. It has also shown good criterion related validity, with the negative self-belief scale being strongly associated with depressive symptoms, and the assumptions subscales with ED-related symptoms (Cooper et al., 1997).

The Ruminative Response Scale (RRS; Nolen-Hoeksema & Morrow, 1991) is a 22-item instrument that assesses the tendency to ruminate in response to low mood. Items are scored from 1 to 4, total scores can thus range from 22 to 88. The RRS has demonstrated good reliability and validity as a measure of rumination (Nolen-Hoeksema & Morrow, 1991).

The Why Ruminate Scale (WRS; Watkins & Baracaia, 2001) is a questionnaire that assesses beliefs about the utility of rumination. It is comprised of 30 items reflecting reasons for rumination, including its use to gain insight into self, solve problems, reduce discomfort, maintain goals, help cope with difficult events and provide direction. Items are rated on a five-point scale. Total scores can range from 30 to 150. Watkins and Baracaia (2001) found that scores on this scale were significantly correlated with self-reports of rumination in a group of dysphoric participants. Barnhofer et al. (2006) report a Cronbach’s alpha of .92.

The Acceptance and Action Questionnaire (AAQ; Hayes et al., 2004) is a 9-item measure of experiential avoidance. Items are rated on a 7-point scale and total scores range from 9 to 63, with higher scores reflecting high experiential avoidance. Studies have reported adequate internal consistency (Cronbach’s alpha = .70), good divergent and convergent validity with measures of thought suppression and avoidant coping (Feldner, Zvolensky, Eifert, & Spira, 2003; Gratz, Rosenthal, Tull, Lejuez, & Gunderson, 2006; Hayes et al., 2004).

### Procedure

The survey was advertised at Oxford University colleges and at various university locations. After obtaining informed consent, participants completed the questionnaires in the order they were described above. Upon completion of the survey, participants were fully debriefed.

### Data analysis

Data were analysed by a series of correlation and regression analyses using an alpha level of .05.

## Results

### Demographics

The mean EDE-Q global score was 1.33 (*SD* = 1.03), which is lower compared to female undergraduate norms (*M* = 1.74, *SD* = 1.30) reported by Luce, Crowther, and Pole (2008), and by Fairburn and Beglin (1994) for a community sample of young women (*M* = 1.55, *SD* = 1.21). While EDE-Q profiles are known to vary between samples and sites, the most likely reason for this difference is that the current study included both males and females. In fact, mean EDE-Q global scores differed significantly between males and females in the current sample (*M* = .79, *SD* = .80 vs. *M* = 1.58, *SD* = 1.12, *p* < .01). Gender was thus controlled for in all analyses. Mean depression and anxiety scores were 5.63 (*SD* = 4.52) and 4.16 (*SD* = 4.07) respectively, indicating mild levels in our sample on the whole. There were no differences between males and females in depression or anxiety levels (*p* = .86 and *p* = .70, respectively).

### Correlations

Partial correlations, controlling for gender, were performed to examine the relationships between study variables of interest. In line with hypotheses, there were significant correlations between EDE-Q scores and all outcome variables: rumination (*r* = .34, *p* < .001), beliefs about rumination (*r* = .31; *p* < .001), experiential avoidance (*r* = .38, *p* < .001), and the number of positive completions on the ED-SCT (*r* = .47, *p* < .001). We also examined correlations between ED-levels and reflection and brooding subscales of the RRS (see Treynor, Gonzalez, & Nolen-Hoeksema, 2003), as previous research has documented differential relations of these subscales with psychopathology (e.g., Crane, Barnhofer, & Williams, 2007; Treynor et al., 2003). This analysis showed significant correlations between EDE-Q scores and both scales: reflection (*r* = .16, *p* = .03) and brooding (*r* = .42, *p* < .001).

### Regression analyses

A series of hierarchical linear regression analyses were carried out to examine the impact of ED-symptomatology on rumination, beliefs about rumination, experiential avoidance, and schematic thinking. Depression, anxiety and gender were entered as covariates at Step 1 of the analyses. EDE-Q scores were entered at Step 2 (see Table 2 for a summary of the regression models).

For rumination, the model was significant at Step 1. Depression and anxiety scores significantly predicted rumination, while gender did not. Entering EDE-Q scores at Step 2 did not improve the overall model.

For brooding, the model was significant at Step 1. Both depression and anxiety scores significantly predicted brooding levels, whereas gender did not. At Step 2, there was a significant improvement in the model, accounting for 43% of variance in brooding scores. EDE-Q was a significant predictor of brooding.

Regression analysis for reflection showed that the model was significant at Step 1 when entering the covariates, with gender and depression scores making significant contributions. Anxiety was not a significant predictor of reflection scores. Entering EDE-Q scores did not improve the model.

For beliefs about rumination, entering the covariates made a significant contribution to the model, with anxiety being a significant predictor of WRS scores. The contribution of depression was at trend-level, while gender was not a significant predictor. At Step 2, there was a significant improvement in the model, accounting for 16% of variance in WRS scores, with EDE-Q entering as a significant predictor.

For experiential avoidance, the model was significant at Step 1 when the covariates were entered, with depression and anxiety being significant predictors of AAQ scores, while gender was not significant. At Step 2, there was a significant improvement in the model, accounting for 36% of variance in AAQ scores, and EDE-Q being a significant predictor of experiential avoidance levels.

For the ED-SCT, the regression model was significant at Step 1, with depression entering as a significant predictor, while anxiety and gender were non-significant. At Step 2, EDE-Q was a significant predictor of ED-SCT and significantly improved the model, accounting for 23% of ED-SCT variance (see Table 2).

We also carried out correlations between the ED-SCT and EDBQ-subscales assessing ED-related assumptions in order to confirm that our new task was assessing interpretative tendencies seen as dysfunctional in the context of ED. As expected, this analysis revealed significant correlations between the number of positive completions on the ED-SCT and all EDBQ-subscales: acceptance by others (*r* = .52, *p* < .001), self-acceptance (*r* = .49, *p* < .001), and control over eating (*r* = .42, *p* < .001).

### Additional analysis

As the outcome variables of the regression analyses above were positively correlated with each other and because this may contribute to their relationship with ED psychopathology, we carried out a separate regression analysis. In this analysis, we entered EDE-Q as the dependent variable and the other factors as independent variables (controlling for depression, anxiety and gender) in an attempt to determine which of above relationships with ED symptoms were maintained after controlling for each other. The results confirmed the significant relationships of EDE-Q levels and ED-SCT completions (*β* = .27, *p* < .001), and EDE-Q and brooding scores (*β* = .42, *p* < .01), as well as EDE-Q and AAQ scores (*β* = .16, *p* = .03). However, the relationship between EDE-Q and WRS scores was no longer significant (*β* = .10, *p* = .18).

## Summary of findings

Findings from Study 1 largely supported our hypotheses. The level of ED-concerns was significantly correlated with rumination, beliefs about rumination, experiential avoidance, and the number of positive ED-SCT completions. Moreover, regression analyses supported the hypotheses that ED-levels would significantly predict experiential avoidance and number of positive ED-SCT completions after controlling for effects due to depression, anxiety and gender. Contrary to predictions, ED-symptoms were not a unique predictor of rumination, which was more closely related to depression and anxiety, and the relationship with beliefs about rumination may have been due to inter-correlations between predictor variables (as suggested by the additional analysis). Analysing the RRS subscales of reflection and brooding showed that ED-scores significantly predicted brooding, but not reflection.

The current findings were based on a non-clinical student sample and thus generalisations to clinical populations of ED were limited. The second study aimed to overcome this by providing some data from a clinical sample.

## Study 2

The aim of Study 2 was to extend findings from Study 1 to a clinical sample. We recruited a small number of patients with anorexia nervosa (AN) and matched controls for this purpose. Our predictions were as follows:

*Hypothesis 1*: We predicted that patients with AN would provide more positive completions on the ED-SCT compared to controls.

*Hypothesis 2*: We predicted that patients with AN would show higher levels of rumination, beliefs about the benefits of rumination, and experiential avoidance than control participants.

We predicted significant differences in rumination and beliefs about the benefits of rumination despite the inconclusive pattern in Study 1, as this could have also been due to the relatively low EDE-Q sample mean. Findings from Study 1 did however lead us to expect a more robust difference between AN patients and controls for ruminative brooding than for reflection.

## Method

### Participants

Fifteen inpatients with AN were recruited from the tertiary ED service for Oxfordshire and Buckinghamshire. Two patients had to be excluded because their EDE-Q scores were outliers and skewed the distribution (defined as at least two standard deviations below the mean). All patients had a diagnosis of AN on admission, as verified by independent clinical examination. The Mini International Neuropsychiatric Interview (MINI; Sheehan et al., 1998) for DSM-IV and the EDE (Fairburn & Cooper, 1993) were carried out to confirm diagnoses. Thirteen student volunteers from the University of Oxford were recruited as controls. Controls had no current physical illnesses and were un-medicated at the time of testing. Other exclusion criteria for this study were active psychosis, any medical condition that significantly affected concentration, history of head injury or stroke, and any physical disability that impacted on body image. Groups were matched in terms of age, gender (all participants were female), and verbal-IQ (see Table 3). Age ranged from 18 to 51 (*M* = 26.08, *SD* = 6.95). Participants received book vouchers for participation.

### Measures

#### Clinical interview

The EDE (Fairburn & Cooper, 1993) is a semi-structured interview that yields both diagnostic and specific psychopathology severity ratings for ED and is the ‘gold standard’ interviewer-based measure of ED psychopathology (Shafran & Fairburn, 2002). It was used to verify diagnosis of ED in the patient group. The EDE has good internal consistency, convergent and divergent validity, test re-test and inter-rater reliability (e.g., Fairburn & Cooper, 1993; Rizvi, Peterson, Crow, & Agras, 2000).

#### Self-report

The BDI and the BAI were used to control for depressive and anxiety symptoms respectively (Beck et al., 1961, 1988). These 21-item self-report scales measure the presence and severity of depression and anxiety symptoms over the previous week. Their validity and reliability have been well established (Beck et al., 1988).

The EDBQ, ED-SCT, AAQ and RRS were described in Study 1. Inter-rater agreement analysis for the ED-SCT showed an overall agreement of 96% between raters and a kappa value of .93.

#### Intelligence

The National Adult Reading Test (NART; Nelson, 1982) requires participants to read aloud 50 phonetically irregular words of increasing difficulty. The NART correlates significantly with scores on full scale, verbal and performance intelligence tests (Nelson & O’Connell, 1978), and was used to match the groups in terms of intellectual ability.

#### Body Mass Index

All participants had their weight and height recorded and their BMI calculated [weight (kg)/height^2^ (m^2^)].

### Procedure

All participants were tested individually. After providing informed consent, clinical interviews were carried out. The EDE was administered by the experimenter who had received training in its use. Height and weight were recorded during the MINI. Subsequently, the questionnaire materials were completed, the order of which was counterbalanced. The study required about 30 min. At the end, participants were debriefed and thanked for participation.

### Data analysis

Data were analysed by a series of independent samples *t* tests. An alpha level of .05 was used for all statistical tests.

## Results

### Sample comparison

The groups were comparable in terms of age and their predicted verbal-IQ (*t*s < .83, *p*s > .41). As expected, the AN patient group showed higher levels of ED pathology than controls on the EDE-Q global scale, *t*(1,24) = 10.95, *p* < .001, *d* = 4.48, as well as all four EDE-Q subscales (*ts*(1,24) > 7.72, *ps* < .001, *ds* > 3.09; note that subscale means are not reported in the table). The AN group had a lower average BMI than controls, *t*(1,24) = 4.27, *p* < .001, *d* = 1.71, greater levels of depression, *t*(1,24) = 6.70, *p* < .001, *d* = 2.68, and anxiety, *t*(1,24) = 3.35, *p* < .001, *d* = 1.34; see Table 3.

### Comparison on outcome variables

As predicted, results confirmed significant differences on the ED-SCT, *t*(1,24) = 5.82, *p* < .001, *d* = 2.08, where the AN group provided more positive completions than the control group. The number of positive completions on the ED-SCT was significantly correlated with EDBQ-subscales: acceptance by others, *r* = .78, *p* < .001, self-acceptance, *r* = .80, *p* < .001, and control over eating, *r* = .72, *p* < .001.

Significant differences were also evident for rumination, *t*(1,24) = 4.10, *p* < .001, *d* = 1.64, beliefs about the benefits of rumination, *t*(1,24) = 6.45, *p* < .001, *d* = 2.58, and experiential avoidance, *t*(1,24) = 7.40, *p* < .001, *d* = 2.96. We also analysed differences on RRS subscales: reflection and brooding. While there was no significant difference between the groups in levels of reflection, *t*(1,24) = 1.31, *p* = .20, there was a significant difference in brooding scores, *t*(1,24) = 4.48, *p* < .001, *d* = 1.83.

As can be seen from Table 3, all these effects were in the expected direction. Moreover, effects remained significant when controlling for BDI, BAI and BMI scores (all *F*s > 5.41, *p*s < .04, *η*_p_ > .20), and adjusting for Bonferroni corrections.

### Additional analysis

In order to provide preliminary data on whether changes in ED-symptom levels were associated with changes in rumination, experiential avoidance and dysfunctional attitudes, AN patients were followed-up approximately 10 months after initial participation for a second assessment of these variables. Although the sample size was small this seemed warranted and potentially informative, as direct evidence showing that improvement in ED-state may be associated with reductions in avoidance and rumination is lacking (Schmidt & Treasure, 2006).

Results showed that EDE-Q change was positively correlated with change in rumination (*r* = .51, *p* = .04), experiential avoidance (*r* = .59, *p* = .02), and the number of positive ED-SCT completions (*r* = .47, *p* = .05). EDE-Q change also correlated with BDI change (*r* = .56, *p* = .02) and brooding change (*r* = .68, *p* < .001), while the correlation with reflection change was at trend-level (*r* = .46, *p* = .06). When partial correlations, controlling for BDI change (EDE-Q change was not associated with BAI or BMI change) were carried out, only the correlations between EDE-Q change and ED-SCT change, and EDE-Q change and brooding change remained significant.

## Summary of findings

The aim of this study was to extend findings from Study 1 to a clinical sample. The results supported our hypothesis of higher levels of rumination, experiential avoidance, and beliefs about the benefits of rumination in AN patients. Consistent with predictions, the patient group also showed greater levels of ED-related underlying assumptions as assessed with the ED-SCT. Analysing RRS subscales revealed that AN patients scored significantly higher than controls on brooding but not reflection. This study only included a small tertiary sample of patients with AN, so cannot be regarded as representative of all cases of ED. However, although the sample was small, the effect sizes were large. Furthermore, all effects held when controlling for levels of depression, anxiety and BMI. Results from the follow-up suggested that changes in ED-symptom level across time were correlated with changes in outcome variables, particularly brooding and the number of positive completions on the ED-SCT, as these relationships remained significant when controlling for changes in depression levels. Due to the limited information obtained between assessment points (e.g., information on life events and treatment status was not available), and the fact that changes in ED-state were not verified by more objective means, findings from this follow-up have to be treated as preliminary. Generally, findings need replication and extension.

## General discussion

The current studies examined aspects of cognitive-affective content and processes in clinical and non-clinical samples with ED pathology. Across both studies, ED-concerned individuals provided a higher number of positive completions on the newly devised ED-SCT, a task assessing the endorsement of dysfunctional attitudes about eating, weight and shape control. This finding suggests that such aspects are more pertinent for ED-concerned individuals compared to those with mild or no ED-concerns. The significant correlations between the ED-SCT and the EDBQ-subscales support the view that the number of positive completions is likely to reflect the extent to which concepts of self and those relating to eating, weight, and shape are inter-related, consistent with the presence of dysfunctional schemas in ED which are likely to have cognitive, emotional and behavioural consequences. One such consequence is that ED-related issues are likely to play a more central role to these individuals’ sense of self with correspondingly global repercussions. Our results suggest that ED-concerned individuals are more likely than others to interpret weight gain in terms of widespread personal inadequacies, whereas successful control provides a general sense of accomplishment, acceptance, and direction.

While preliminary, results from the follow-up supplement these findings pointing to the presence of dysfunctional cognitions, by suggesting changes in aspects of schematic thinking with changes in ED-symptomatology. Such changes are likely to imply a contextual alteration of the relationships between constructs of self and ED-related issues, whereby their inter-relationships and thus personal significance become less prominent as eating behaviour begins to normalise. As a result, personal well-being is not as extremely dependent on success or failure in ED-related domains, which is reflected in a change in the number of positive ED-SCT completions.

The ED-SCT could be a useful tool to assess both the *presence* and *changes* in aspects of schematic thinking in experimental and clinical studies. The ED-SCT showed significant correlations with EDBQ-subscales, was related to current ED-symptoms and changes over time (independent of depression and anxiety), and distinguished ED-concerned individuals from controls. Furthermore, it is brief and requires the generation of own responses rather than merely rating agreement or not, so may be more indicative of unique schematic content. Valence or latency ratings of completions may be a suitable addition in this regard and enable a more fine-grained analysis of responses. Moreover, it seems worthwhile to investigate differences in functional in addition to dysfunctional completions (e.g., absence of the latter does not necessarily imply ‘access’ to functional interpretations), and how the ED-SCT relates to the persistence of ED-related goals.

The above finding is also of theoretical relevance for the way schemas are conceptualised. Whereas the traditional Beckian view of schemas adopted by most cognitive theories in ED regard these as relatively enduring propositional rules or beliefs which constitute a vulnerability factor (Beck, 1976), other researchers such as Teasdale and Barnard (1993) use the term schematic models to encapsulate cognitive representations that are more contextually-based and dynamically changing. Teasdale and Barnard hypothesise that recovery from psychopathology would be associated with changes in the mental models that become ‘switched in’ and through which experience is interpreted. Our findings are more consistent with the view that changes in ED-related thinking may be the result of changes of dynamic schematic models.

As argued earlier, the presence of persistent disorder-specific thoughts is likely to elicit further cognitive processing (Hayes et al., 2004). As such the interplay between content and process may be crucial for understanding of psychopathology, and Teasdale and Barnard (1993) have argued that schematic models are maintained by specific patterns of information processing.

Our findings suggested that information processing in ED is likely to be characterised by rumination, particularly brooding, and avoidance of internal states and experiences. Studies 1 and 2 supported this proposition, showing an association of ED psychopathology with ruminative brooding and with experiential avoidance (Study 1), and differences between patients with AN and controls (Study 2). While findings were mostly consistent with hypotheses, results for rumination were not fully conclusive (e.g., ED pathology did not significantly predict rumination levels). It is possible that this relationship becomes more apparent with greater pathology (Study 2 did find significant differences between AN patients and controls) but this could also be at least partly explained by the rumination scale employed. The RRS assesses rumination in the context of low mood and, indeed, data suggested strong relations with depression. This suggests that assessment of this process will be enhanced by the use of context independent rumination scales (e.g., the Perseverative Thinking Questionnaire; Ehring, 2007), or those that assess rumination on ED-specific content. While there is likely to be overlap between the content of ruminative processing in ED and depression, there are also important differences, as much thinking in ED focuses on perceived self-discrepancies and imperfections over control of eating, weight, and shape. Another characteristic feature of ED is the ambivalent and at times positive attitude towards some aspects of the disorder (e.g., Serpell et al., 1999). Thus, amendments to current measures will permit more accurate assessment of the extent and characteristic nature of ruminative thinking in ED.

On the whole, it seems likely that rumination and avoidance are relevant features of mental processing activity in ED, which is in accord with the evidence to date that suggests a relationship to abnormal eating patterns/attitudes (e.g., Nolen-Hoeksema et al., 2007), and the proposition that they are transdiagnostic processes. Thus, their consequences are likely to be similar to those in other disorders, particularly over time. Information processing strategies characterised by brooding and experiential avoidance are related to the persistence of symptoms, unachieved goals and maladaptive emotion regulation (Burwell & Shirk, 2007; Hayes et al., 2004; Treynor et al., 2003), whereas reflective processes are associated with insight, adaptive coping strategies, and behaviour change (although this latter evidence is not without qualification, see Surrence, Miranda, Marroquin, & Chan, 2009).

Rumination may be seen as a pertinent strategy for maintaining focus on goals and control over thoughts and feelings despite its objective negative consequences. This view of the potential function of rumination received substance from findings of greater beliefs about the benefits of rumination in AN patients compared to controls. Feelings of control are particularly important in ED (Fairburn et al., 1998). Moreover, ruminating on *ideas* of self and body reduces their direct *experience*, and rumination may thus be viewed as beneficial by patients with ED where overcoming emotional and bodily sensations such as cold, hunger, or pain seems crucial for continued dietary restraint (Park et al., under review).

Recent theories have begun to consider the processes that underlie cognitive-affective content (Waller et al., 2007; Wolff & Serpell, 1998). Park et al. (under review) suggest that rumination and avoidance are features of a distinct pattern of processing activity (an analytical mode of processing), which dominates the subjective experience of AN and contributes to the maintenance of dysfunctional schematic representations. Their analysis suggests that the *way* thoughts and feelings about self and body are processed may have causal effects on symptoms. Research in depression has demonstrated that the specific mode of processing self-referent information has distinct effects on measures of emotion and cognition (e.g., Watkins, Moberly, & Moulds, 2008; Watkins & Teasdale, 2001, 2004). This work underpinned interventions that target the mode of processing for facilitating recovery and preventing relapse (Rawal, Enayati, Williams, & Park, 2009; Segal, Williams, & Teasdale, 2002; Watkins & Moberly, 2009).

Extrapolation of follow-up data from Study 2 suggests that recovery from AN may be associated with reductions in ruminative processes (particularly brooding) and experiential avoidance (although this conclusion was limited by the small sample and statistical constraints). The functions and effects of such cognitive processes, their interactions, as well as the interplay with disorder-specific content require further evaluation. Their study is likely to aid understanding of development and maintenance of psychopathology. For example, research in depression suggests that rumination may predict the onset, whereas the interaction of rumination and negative cognitive styles predicts the duration of depressive symptoms (see Nolen-Hoeksema et al., 2008). Systematic longitudinal studies are needed to provide insight into these processes and interactions in ED.

Experimental investigations testing the causal influences of processes on symptoms and self-regulation (e.g., Rawal, Park & Williams, in preparation) are also likely to provide a clearer picture as to their implications for the maintenance of ED. The current studies do not enable any causal inferences due to their non-experimental design. Furthermore, the sample did not include a depression only or dieting control group. The latter would have enabled conclusions about differences in schema-content, rumination, and experiential avoidance due to ED-related factors other than dieting. Future studies should also employ more objective measurements of the processes we intended to assess, as our methods solely relied on self-report. The inclusion of thought sampling assessments or physiological indices of repetitive thought would be a valuable inclusion, possibly also for teasing the influence of different cognitive processes further apart.

In sum, findings from the present studies suggest that individuals with ED psychopathology are distinguishable from controls in both the cognitive-affective content and the processes by which they are likely to respond to such concerns. While cognitive theories and treatment of ED have mainly focused on specifying and dealing with disorder-specific thoughts, studying the mental processing activity underlying such information may advance understanding of the maintenance of ED psychopathology and suggest ways for improving emotion regulation. Such a trend would follow theory and research in other psychopathologies where a process-focused approach has led to important insights in the understanding of psychopathology and the delivery of treatment (Teasdale, 1999; Williams, 2008).

## Figures and Tables

**Table 1:** ED-SCT stems with typical ‘functional’ and ‘dysfunctional’ completions.

Sentence stem	‘Functional’ completion	‘Dysfunctional’ completion
If I could always be my ideal weight and shape then others would ….. me.	‘envy’	‘like’
For everyone to look to me for guidance and advice about how to keep trim and slim would make me ……	‘annoyed’	‘happy’
If I always go out of my way to look perfect, people will ….. me.	‘dislike’	‘respect’
By slave-driving my body I will make myself ……	‘ill’	‘better’
Putting all my effort into attaining my ideal weight is the way to ……	‘unhappiness’	‘happiness’
Always seeking to meet cultural ideals of beauty is the road to ……	‘ruin’	‘happiness’
Aiming to impress others with your slim, perfect shape is a good way to make them …… you.	‘hate’	‘like’
If you take great care never to eat unhealthy fattening foods people will see you as ……	‘obsessive’	‘healthy’
Placing great importance on my success at being thin is likely to prove ……	‘nothing’	‘successful’
If I demand perfection of my body I will make myself ……	‘ill’	‘better’
Never breaking my diet plan means I will be ……	‘bored’	‘strong’
Always to put self-control before your desires is a recipe for ……	‘disaster’	‘success’

**Table 2:** Regression models examining contribution of gender, depression, anxiety, and eating disorder psychopathology on prediction of outcome variables for Study 1 (*N* = 177).

Block	Variable	*B*	*SE B*	*β*	*t*	*p*	∆*R*^2^	∆*F*
Model 1	RRS total					<.001	.44	45.32
	Gender	.10	.12	.05	.82	.41		
	Depression	.53	.09	.47	5.93	<.001		
	Anxiety	.26	.09	.23	2.95	<.01		
Model 2	ED concern	.07	.07	.06	.93	.35	<.01	.87
Model 1	RRS Brooding					<.001	.40	38.54
	Gender	−.01	.43	−.001	.02	.98		
	Depression	.30	.06	.40	4.92	<.001		
	Anxiety	.23	.07	.28	3.42	<.01		
Model 2	ED concern	.63	.21	.21	2.99	<.01	.03	8.93
Model 1	RRS Reflection					<.001	.17	11.50
	Gender	.31	.15	.14	2.06	.04		
	Depression	.28	.11	.24	2.51	.01		
	Anxiety	.17	.11	.15	1.54	.13		
Model 2	ED concern	.01	.09	.01	.07	.94	<.001	.01
Model 1	WRS					<.001	.13	8.53
	Gender	−.03	.15	−.01	.19	.85		
	Depression	.22	.11	.19	1.92	.06		
	Anxiety	.23	.11	.20	2.03	.04		
Model 2	ED concern	.24	.09	.22	2.56	.01	.03	6.53
Model 1	AAQ					<.001	.34	29.39
	Gender	−.08	.13	−.04	.63	.53		
	Depression	.35	.10	.31	3.57	<.001		
	Anxiety	.37	.10	.33	3.78	<.001		
Model 2	ED concern	.21	.08	.19	2.60	.01	.03	6.74
Model 1	ED-SCT					<.001	.11	6.78
	Gender	−.04	.16	−.02	.23	.82		
	Depression	.40	.12	.34	3.37	<.01		
	Anxiety	−.02	.12	−.02	.18	.86		
Model 2	ED concern	.49	.09	.43	5.31	<.001	.13	28.17

*Note*. RRS = Ruminative Response Scale; WRS = Why Ruminate Scale; AAQ = Acceptance and Action Questionnaire; ED-SCT = Eating Disorder-Sentence Completion Task.

**Table 3:** Sample characteristics and comparison between patients with anorexia nervosa and healthy controls on outcome variables for Study 2.

Variable	AN patients (*N* = 13)		Controls (*N* = 13)	
	*M*	*SD*	*M*	*SD*
Age	26.38	8.77	25.77	4.85
EDE-Q Global	4.37	1.17	.53	.47
BMI	17.16	1.61	21.06	2.87
BDI	28.92	13.33	3.92	1.85
BAI	21.92	13.77	7.00	8.29
NART	119.85	3.48	121.00	3.70
ED-SCT	7.77	3.14	2.08	1.61
RRS Total	59.23	10.89	41.85	10.72
RRS Reflection	13.38	3.71	11.38	4.07
RRS Brooding	14.62	2.29	9.54	3.38
WRS	93.85	21.52	50.77	10.84
AAQ	48.15	7.29	30.23	4.82

*Note*. EDE-Q = Eating Disorder Examination-Questionnaire; BMI = Body Mass Index; BDI = Beck Depression Inventory; BAI = Beck Anxiety Inventory; NART = National Adult Reading Test; ED-SCT = Eating Disorder-Sentence Completion Task; RRS = Ruminative Response Scale; WRS = Why Ruminate Scale; AAQ = Acceptance and Action Questionnaire.
